# Colonic Epstein-Barr virus mucocutaneous ulcer mimicking metastatic cancer: a diagnostic pitfall on PET/CT in immunosuppressed patients

**DOI:** 10.1093/bjrcr/uaaf058

**Published:** 2025-12-02

**Authors:** Nathan Truong, Christopher Henderson, Michael Lin

**Affiliations:** Department of Nuclear Medicine and PET, Liverpool Hospital, Sydney, NSW 2170, Australia; Department of Anatomical Pathology, New South Wales Health Pathology, Liverpool Hospital, Sydney, NSW 2170, Australia; School of Medicine, Western Sydney University, Sydney, NSW, 2560, Australia; South Western Sydney Clinical School, UNSW Medicine, Sydney, NSW, 2170, Australia; Department of Nuclear Medicine and PET, Liverpool Hospital, Sydney, NSW 2170, Australia; School of Medicine, Western Sydney University, Sydney, NSW, 2560, Australia; South Western Sydney Clinical School, UNSW Medicine, Sydney, NSW, 2170, Australia

**Keywords:** [18F]-FDG-PET/CT, Epstein-Barr virus, mucocutaneous ulcer, colon cancer

## Abstract

Epstein-Barr Virus-positive mucocutaneous ulcers (EBVMCU) are a rare disease entity that causes mucocutaneous ulcerations in the gastrointestinal tract, oropharynx, and skin. Typically associated with immunosuppressed patients, individuals may present with nonspecific symptoms and scan findings similar to those of malignancies. Treatment usually responds favourably to conservative management or withdrawal of immunosuppression, although rarely patients require more aggressive therapies including surgery, immuno-, chemo-, or radio-therapy. We encountered an unusual case of a patient with EBVMCU who presented with cough, dysphagia, and weight loss. Imaging features on [18F]-FDG PET/CT as well as initial biopsy results were nonspecific. The colonoscopic features were highly concerning for malignancy, with the diagnosis only finally being confirmed following surgical resection. To our knowledge, this is the first case report to describe the FDG PET/CT findings of EBVMCU within the bowel, and readers should consider this as a differential to avoid potential misdiagnosis and unnecessary intervention.

## Background

[18F]-Fluorodeoxyglucose (FDG) positron emission tomography/computed tomography (PET/CT) is well established in the assessment of malignancy. Unexpected focal FDG uptake in the large bowel on PET/CT is not uncommon, and approximately 50%-80% of cases are due to a dysplastic polyp or malignancy.^1^ However, not all FDG-avid lesions are neoplastic, and certain inflammatory or infectious conditions can present similarly on imaging. Epstein-Barr Virus-positive mucocutaneous ulcer (EBVMCU) is a newly recognized clinical entity that causes unifocal, well-circumscribed ulcerations in the mucosal and cutaneous surfaces of the gastrointestinal tract, oropharynx, and skin.^2^ In most patients, EBVMCU follows an indolent course that responds well to conservative management.^3^ We report a case of EBVMCU mimicking a neoplastic bowel lesion on PET/CT, leading to surgical resection.

## Case presentation

**Figure 1.**
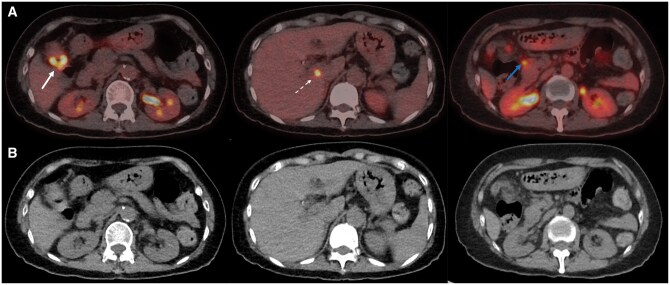
(A) Axial fused [18F]-FDG PET/CT abdomen, and (B) axial low-dose CT. The images demonstrate intense FDG uptake within the hepatic flexure (solid white arrow) as well as intense hypermetabolic activity in the caudate of the liver (broken white arrows), and moderate uptake in an adjacent pericolic lymph node (solid blue arrow).

A 70-year-old female with CREST (Calcinosis, Raynaud’s phenomenon, Oesophageal dysfunction, Sclerodactyly, Telangiectasis) syndrome presented to the Immunology Clinic with worsening reflux and cough. Her major manifestations included scleroderma-associated interstitial lung disease, oesophageal dysmotility, and Raynaud’s phenomenon. She was a previous bone marrow transplant recipient and was taking regular mycophenolate. A non-contrast high-resolution CT scan of the chest was obtained to assess for progression of pulmonary fibrosis and showed interval growth of a right lung nodule. A subsequent [18F]-FDG PET/CT scan for further characterization revealed mild FDG-uptake in the nodule (maximum standardized uptake value [SUVmax] 2.1). Incidental irregular, intense focal uptake (SUVmax 12.4) was also observed in the hepatic flexure of the colon, with adjacent moderately FDG-avid abdominal lymph nodes and intense focal uptake in the caudate lobe of the liver (Figure 1). Given the radiological pattern of locoregional nodal and hepatic involvement, a provisional diagnosis of metastatic colon cancer was raised.

**Figure 2.**
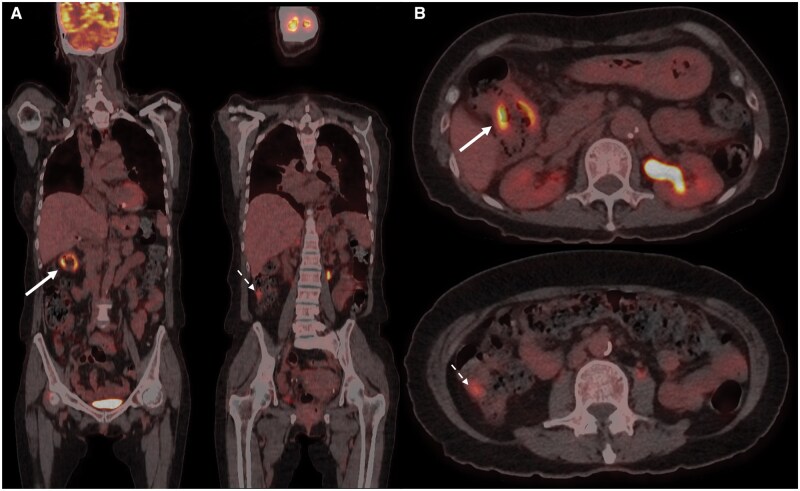
Repeat [18F]-FDG PET/CT performed 6 months later with (A) coronal fused PET/CT, (B) and axial fused PET/CT showed persistent, intense uptake at the hepatic flexure (solid arrow) and a new, moderately avid focus more inferiorly (broken arrow). The uptake within the liver and lymph nodes was no longer visualized (not shown).

On further questioning, she reported solid food dysphagia and unintentional weight loss. A faecal occult blood test performed the year prior was negative. She subsequently underwent a colonoscopy, which revealed a solitary ulcer in the descending colon with otherwise normal mucosa elsewhere. Nine biopsies were taken and showed acute inflammatory exudate and areas of ulceration, but no evidence of dysplasia or malignancy. Given that multiple specimens had been obtained, a false-negative result was felt to be less likely and following multi-disciplinary team discussion, a decision was made to continue monitoring the patient. A follow-up PET/CT performed 6 months later showed more extensive and persistently avid uptake at the hepatic flexure (SUVmax 9.9) and a new focal lesion in the proximal ascending colon (SUVmax 5.7) (Figure 2). Interestingly, despite the absence of treatment, the previously demonstrated metabolically active lymph nodes and caudate liver lesion had resolved.

**Figure 3.**
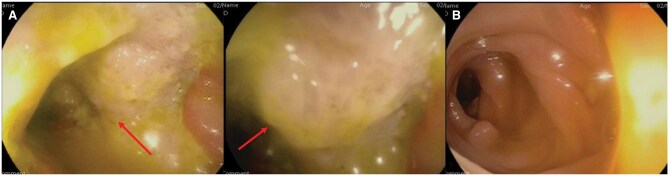
Colonoscopy showing an (A) ulcerated, partially obstructing mass at the hepatic flexure (solid arrows) with (B) normal transverse colon mucosa for comparison.

**Figure 4.**
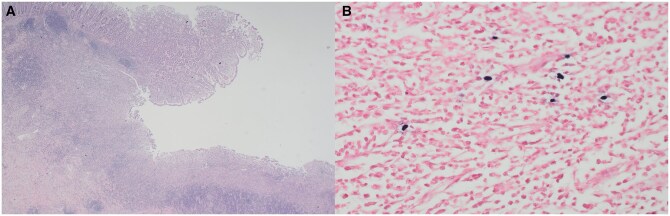
Haematoxylin and eosin-stained slides with (A) low-power microscopy showing ulcer of colon down to submucosa with the base lined by heavy lymphoplasmacytic infiltrate, (B) High power showing scattered small to large EBV-positive lymphoid cell nuclei within the heavy lymphoplasmacytic infiltrate of the ulcer.

A repeat colonoscopy was undertaken and revealed a 3 cm ulcerated, partially obstructing mass at the hepatic flexure, with histology again demonstrating acutely inflamed granulation tissue with necroinflammatory slough (Figure 3). Given the concerning macroscopic appearances, a decision was made to proceed to operative management and 2 months later, she underwent a laparoscopic right hemicolectomy. Histopathological analysis of the resected specimen confirmed EBVMCU, with no evidence of malignancy (Figure 4). The patient recovered well following surgery and, despite continuing her regular dose of mycophenolate (1 g twice daily), had satisfactory resolution of symptoms.

## Differential diagnoses

Lymphoma and cytomegalovirus (CMV) infections were also key differential considerations in this context; however, both T-cell receptor (TCR) and immunoglobulin (Ig) gene clonality studies were negative, immunoglobulin light chain in situ hybridization showed no light chain restriction, and CMV immunohistochemistry was negative.

## Discussion

Epstein-Barr virus, or herpesvirus type 4, is one of the most prevalent viruses globally, infecting over 90% of adults. Transmission typically occurs through exchange of oropharyngeal secretions, but may also occur through sexual intercourse, haematopoietic cell or solid organ transplantation, or blood transfusion.^4^ The oncogenic potential of EBV is well-established and associated with a number of lymphoproliferative disorders, including Burkitt’s lymphoma, classic Hodgkin’s lymphoma, and post-transplant lymphoproliferative disease.^4^ Among these is EBVMCU, a recently described pathological entity characterized by Dojcinov et al. in 2010, and later included in the 2016 WHO classification of haematopoietic and lymphoid tumours.^2^^,^^3^

EBVMCUs typically involve mucocutaneous sites, with common locations including the oropharynx (65.6%), gastrointestinal tract (19.9%), and skin (9.7%), with remaining cases affecting the nasopharynx, sinuses, and gingiva.^3^ Histologically, they are characterized by the presence of polymorphic cellular infiltrate with EBV-positive atypical large B-cell lymphoblasts sometimes resembling a Hodgkin and Reed-Sternberg-like morphology.^2^^,^^3^ Immunosuppression appears to be a key risk factor—whether due to medications, age-related immunosenescence, primary immunodeficiency, HIV/AIDS, or haematopoietic/solid organ transplantation.^3^ While most cases follow a self-limiting course that responds well to conservative management or reduction of immunosuppression, a minority may require more aggressive interventions, including immunotherapy, chemotherapy, radiotherapy, or surgical resection.^3^

The radiological findings of gastrointestinal EBVMCU are poorly defined, with current literature consisting mainly of case reports. Reported CT features include focal bowel wall thickening and lymphadenopathy, which can be inherently non-specific.^5^^,^^6^ Mural thickening alone has limited diagnostic specificity, and differential considerations include malignancy, inflammatory bowel disease (eg, Crohn’s disease, ulcerative colitis), and vascular conditions such as haemorrhage or ischaemia. The severity and distribution of mural thickening can aid in differentiation: infectious or inflammatory conditions often demonstrate symmetric thickening, either focal or diffuse, while malignancy tends to produce focal, asymmetric thickening.^7^ Additionally, perimesenteric fat stranding that appears disproportionately greater than the degree of wall thickening favours an inflammatory or infectious aetiology over malignancy.^7^ The use of intravenous contrast-enhanced CT may provide further utility. The “water halo” sign—an outer hyperattenuating ring surrounding an inner low-attenuation layer of bowel wall—is commonly associated with inflammatory bowel disease, infection, vasculopathy, or radiation enteritis, and is less typical of malignancy.^8^ Variants of this sign include a reversed halo (low-attenuation outer ring and hyperattenuating inner ring) and the “target sign,” which features three concentric layers with alternating low- and high-attenuation patterns.^8^

On [18F]-FDG PET/CT, differentiating between infectious and malignant conditions can be challenging since there is no absolute SUV cutoff to distinguish between the two. Significant overlap in FDG uptake levels exists between benign and malignant disease, and certain neoplasms—such as mucinous adenocarcinomas—may demonstrate low or even absent FDG avidity.^9^ While intense focal colonic uptake would favour a pre-malignant lesion (eg, dysplastic polyp) or malignancy, it can also be seen in a number of benign conditions such as diverticulitis, appendicitis, or abscesses.^9^ As such, image interpretation should incorporate clinical history, oncological risk factors, and corresponding CT features.

In our case, malignancy was initially favoured due to the patient’s constitutional symptoms, irregular bowel wall thickening, and FDG uptake pattern. Particularly concerning were the metabolically active lymph nodes and the intensely avid hepatic lesion—features more commonly associated with metastatic disease and, to our knowledge, not previously described in EBVMCU. However, the patient’s history of immunosuppression, repeated negative biopsies, and spontaneous resolution of FDG-avid lesions without treatment argued against this. This case highlights the importance of considering EBVMCU as a differential diagnosis for focal bowel wall uptake with hepatic and nodal involvement, particularly in immunosuppressed patients.

## Conclusion

EBVMCU, a rare condition seen in immunosuppressed or elderly patients, should be considered as a differential for focal bowel wall uptake and self-resolving lymphadenopathy, given its favourable response to conservative management and immunosuppression withdrawal.

## Learning points

- EBVMCU, though rare, is a potential false-positive that should be considered in immunosuppressed patients with focal bowel wall uptake on [18F]-FDG PET/CT.
- While [18F]-FDG PET/CT is a powerful tool for detecting malignancy, it lacks specificity in distinguishing neoplastic from infectious or inflammatory processes.
- On CT, infectious or inflammatory pathologies may present with symmetric, focal, or diffuse mural thickening, whereas malignancy tends to appear asymmetric and focal.
- The “water-halo” sign on contrast-enhanced CT may be seen in infectious or inflammatory conditions but is less common in malignancy.
